# CPAP (Continuous Positive Airway Pressure) is an effective and stable solution for heart sparing radiotherapy of left sided breast cancer

**DOI:** 10.1186/s13014-020-01505-7

**Published:** 2020-03-06

**Authors:** Aaron M. Allen, Yasmin Korzets Ceder, Tzippy Shochat, Eyal Fenig, Aron Popovtzer, Dimitry Bragilofsky, Adi Alfassy, Helena Allon

**Affiliations:** Department of Radiotherapy, Davidoff Center Rabin Medical Center and Sackler Faculty of Medicine Tel Aviv University, 49 Jabotinksi St, 49100 Petach Tikvah, Israel

**Keywords:** Breast Cancer, Radiotherapy, CPAP, Cardiac toxicity

## Abstract

**Purpose:**

Limiting the heart dose in left sided breast cancer radiotherapy is critical. We sought to study the effect of using CPAP (continuous positive airway pressure) as an aid in reducing heart dose in breast cancer radiotherapy.

**Methods:**

Patients with left sided breast cancer receiving adjuvant radiotherapy were enrolled on a prospective IRB (institutional review board) approved clinical trial utilizing CPAP during radiotherapy. Each patient was simulated and planned with and without CPAP and the best dosimetric results determined the patient’s treatment. Data on the differences in lung and heart volume and position as well as boost cavity position with and without CPAP were analyzed.

**Results:**

Twenty-four women from 10/16 to 10/18 were enrolled. Seven patients were not treated on study; only two of these were due to treatment issues. Median age was 54 years. 70% had breast only radiation and 30% were treated to breast\CW (chest wall) and regional nodes. The median lung volume with CPAP was 60% larger than without CPAP. (1637 vs. 996 cc) *p* < 0.001. The median heart volume decreased 12% with CPAP. (338 vs. 382 cc) In regards to the DVH, CPAP decreased mean heart dose from 3.02 to 1.6Gy (*p* = .0075) and V20 of the lungs from 17.1 to 13.8 with CPAP but this was not significant.

**Conclusion:**

CPAP assisted radiotherapy was tolerable and produced superior treatment plans in left sided breast cancer. This method is worthy of further investigation as a method to normal tissue sparing treatment of left sided breast cancer patients.

## Introduction

Adjuvant radiotherapy for localized breast cancer is a critical part of the management of the most common malignant disease in women worldwide. It has been shown in multiple studies, that left sided radiotherapy with suboptimal techniques has led to increased mortality secondary to cardiac events [1].

Multiple studies have been done using techniques to improve the dosimetry of left sided breast radiotherapy. These include two main solutions: improving dosimetry through treatment planning [2, 3] (IMRT, proton therapy etc.) or distancing the heart physically through technological advances [4, 5] (Automated Breathing Control –ABC, Deep inspiration breath hold (DIBH)). The majority of centers today utilize a technological advance to spare the heart in left sided breast radiotherapy.

Goldstein et al. published a novel technique to improve thoracic radiotherapy utilizing a well-known technology CPAP (continuous positive airway pressure) to improve thoracic dosimetry [6]. Since CPAP has not been extensively studied in breast cancer we chose to examine its use compared to standard of care tangential radiation without breath hold or other techniques. We therefore designed a prospective clinical trial to examine the impact of CPAP during left sided breast cancer radiotherapy and herein report our findings.

## Materials and methods

From 10/2016 to 10/2018, patients with left sided breast cancer requiring adjuvant radiotherapy were enrolled on a prospective phase II trial with IRB approval. Patients were eligible for enrollment following lumpectomy or mastectomy for localized left breast cancer. Patients receiving intraoperative radiotherapy were excluded.

### Study design

This study was designed as a phase II study with the primary endpoint of reduction in mean heart dose with use of CPAP. With an estimated reduction of 1.0 Gy and power of 80% the calculated sample size was 20 patients. Each patient was compared to itself (CPAP vs. No CPAP) which formed the two study groups.

### CPAP training

In order to facilitate the use of daily CPAP assisted radiotherapy patients were trained prior to simulation to wear the CPAP mask and to acclimatize them to positive pressure. Every patient underwent pulmonary function tests and respiratory clearance for CPAP prior to initiation of CPAP. Initial CPAP pressure was chosen at 4 mmHg and gradually elevated per patient comfort to a goal pressure of 15 mmHg.

### Simulation procedure

On the day of simulation patients initially underwent CT simulation (GE lightspeed). Patients were placed supine with breast board elevation as standard in our department for breast radiotherapy.

Patients were scanned initially without CPAP both at free breathing (slice thickness 2.5 mm) as well as utilizing 4D-CT. 4DCT images were obtained with phase gating and reconstructed on the GE Advantage workstation utilizing 10-phase bins.

Following non-CPAP simulation, patients returned to the clinic and were again fitted with their CPAP mask and gradually increased to the target pressure of 15 mmHg.

CPAP assisted simulation was identical to free breathing simulation including initial helical scan followed by 4DCT images obtained with CPAP.

### Radiotherapy planning

Contouring of CTV nodes or breast boost cavity, as well as normal avoidance structures of lungs and heart was done for all patients. Each patient contours underwent 3D-CRT (three dimensional conformal radiotherapy) planning for adjuvant breast cancer as is standard in our department. Typically this utilized two tangential fields with subfields to improve homogeneity and avoid hot spots as is standard in our department. In cases where axillary nodes were treated a single isocenter technique was used for 3 and 4 field plans. IMRT was not used in this study.

### CPAP treatment

Prior to daily treatment with CPAP patients were prepared for their treatment by undergoing a process of acclimatizing their CPAP pressure to the level that it was set to during simulation. After reaching steady state they were transferred to the linac for therapy with CPAP in place. KV images and MV portal images were used for verification. No additional modifications of normal treatment were necessary with CPAP.

### Target coverage

Both CPAP and non-CPAP plans were created with strict criteria of 95% of the prescription dose being delivered to 95% of the breast and nodal (when relevant) PTV.

Normal tissue constraints that were used included:

V20 < 35% (one lung) when nodes were treated and V20 < 15% fr breast alone. Heart constraints were focused on mean heart dose with goals of <4Gy with nodal irradiation and < 3 Gy with breast alone treatment.

Treatment was planned on Eclipse V13.5 (Varian, CA) with a AAA 13.5.35 algorithm.

Following planning on both non-CPAP and CPAP scans the superior plan was chosen for patient treatment.

DVH (dose volume histograms) were calculated for heart and lungs for each patient both with and without CPAP.

### 4DCT analysis

In addition, to comparing free breathing planning with and without CPAP, we sought to analyze the stability of motion during treatment with and without CPAP. To that end we re-contoured both the heart volume as well as the CTV at the extremes of the breathing cycle at the 50% (exhale) and 0% (inhale) states.

These volumes were then analyzed for the mm of overlap between the CPAP (inhale –exhale) and the non-CPAP (inhale to exhale).

### Directional heart movement analysis

In an attempt to analyze the movement of the heart with and without CPAP a vector based on the difference of center of mass was calculated using ECLIPSE (using the Arc Geometry Tool in External Beam Planning). This vector was plotted in a classic three-dimensional coordinate system.

### Statistical analysis

The statistical analysis for this paper was generated using SAS Software, Version 9.4. Continuous variables were presented by Mean ± Std, Categorical variables were presented by number and percentages. Students’ t-test was used to compare univariate variables. Two-sided *p* values less than 0.05 were considered statistically significant.

## Results

### Enrollment

Twenty-four Patients were enrolled on this prospective phase II trial from October 2016 to October 2018. Three patients withdrew consent prior to treatment. One patient had an unknown latex allergy and had a reaction to the CPAP mask. One patient elected to receive treatment at another center closer to her home. One patient refused radiotherapy. Finally one patient began treatment but was unable to tolerate the CPAP mask. The 17 remaining patients that were enrolled and treated and form the basis of this report.

### Patient characteristics

Of the seventeen patients treated, 50% were of Caucasian, and 40% were of Mediterranean descent, 10% were of Arabic descent. Median age was 54 years. Seventy percent were positive for both estrogen and progesterone receptor status. AJCC TNM stage was Stage I in 50, 25% Stage II and remainder had DCIS or Stage III.

### Treatment characteristics

Of the seventeen patients 15% of patients were treated with tangents to breast alone and 55%of patients were treated to breast plus tumor bed boost. 15% of patients received radiation to the breast plus regional nodes and 15% received post-mastectomy radiotherapy to the chest wall and regional nodes.

In the breast only patients the prescription dose was 42.72 Gy in 16 fractions. In the patients receiving regional nodal irradiation the prescribed dose was 50Gy in 25 fractions.

### Dosimetric details

**Fig. 1:**
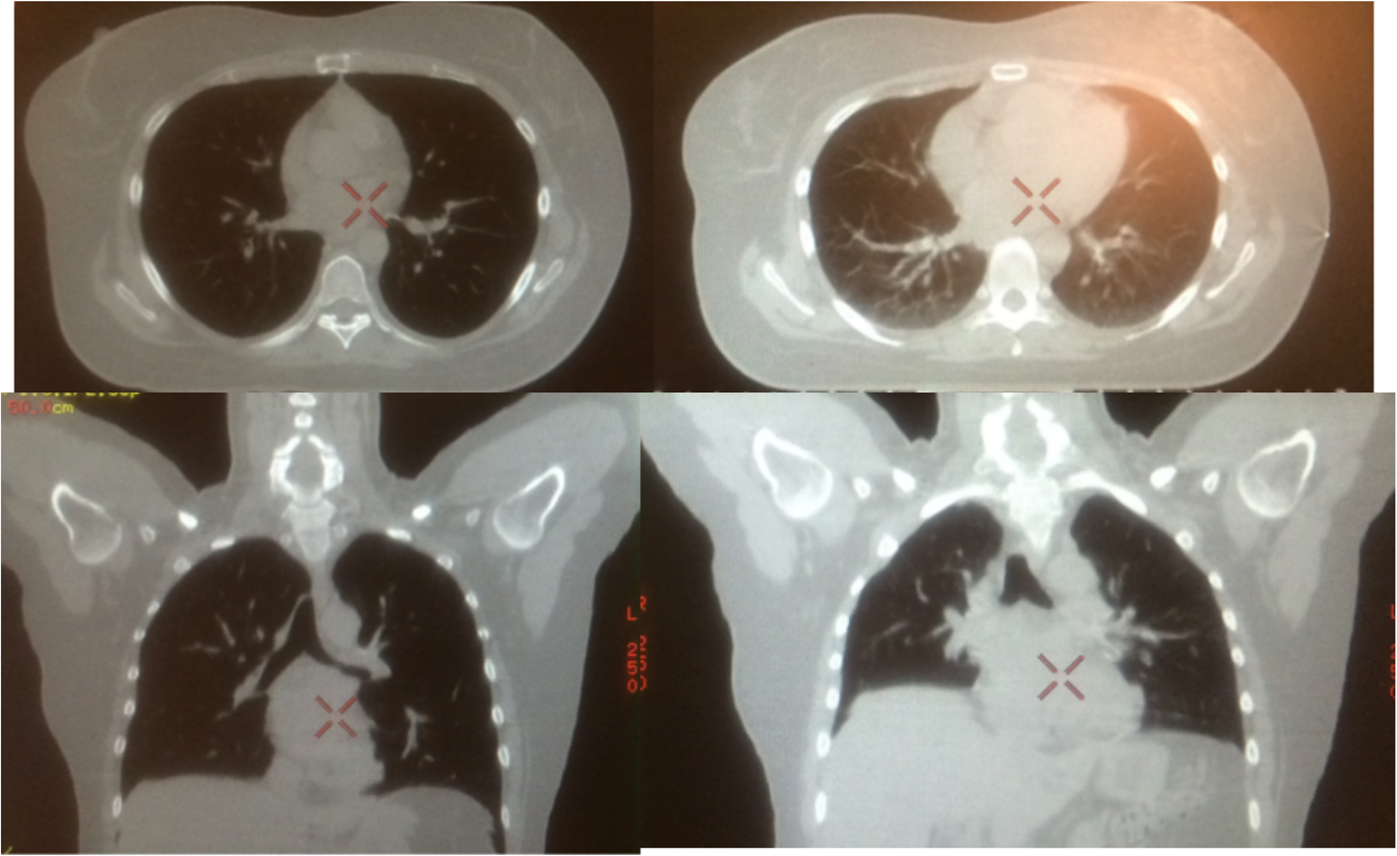
A comparison of patient on study at simulation. The images on the right are at free breathing axial and coronal. The left hand images are with CPAP at 15 mm of pressure showing increased lung volume and significant cardiac shrinkage and displacement

The dosimetric details of the CPAP vs. No CPAP can be divided into change in volume of the organ at risk and the planning or DVH differences based on CPAP or no CPAP. The median lung volume with CPAP was 1637 cc (range 1299–2330) compared to 996 cc (range 706-1474 cc) representing a mean difference of 60% greater lung volume with CPAP (*p*<. 0001). Interestingly the median heart volume with CPAP was 338 cc (range 199–559) as compared to 382 cc (range 232–692) for a decrease of 12% on average with CPAP but this was not statistically significant. A representative case can be seen in Fig. 1. In all cases where plans were compared the CPAP plan was superior in all cases and was used for treatment.

In reference to differences in DVH metrics, the most significant difference was mean heart dose. Without CPAP the mean heart dose averaged 3.02 Gy vs. 1.6 Gy with CPAP (*p* = .0075). Of note there were 5 cases where the mean heart dose with standard planning was above 3.5 Gy. In all of these cases CPAP reduced the mean heart dose to under 3 Gy. The ipsilateral V20 was also reduced from an average of 17.1% without CPAP to 13.8% with use of CPAP but this was not statistically significant. The complete dosimetric results can be seen in Table 1.

**Table 1:** Comparison of dosimetric data in patients’ plans with and without CPAP

	With CPAP Median (SD)	Without CPAP Median (SD)	
Lung Volume (cc)	1506 (348)	945 (220.5)	***p*** **< .0001**
Mean Lung Dose (both lungs)	6.68 Gy (5.8)	6.39 Gy (3.5)	*p* = 0.42
Lung V5	26.3%(11.9)	25.9 (16.6)	*p* = 0.87
Lung V10	15.9%(9.18)	16.4 (14.75)	*p* = 0.41
Lung V20	11.3%(7.7)	11.74 (13.7)	*p* = 0.22
Heart Volume (cc)	351.9 (84.97)	352.8 (122.6)	*p* = 0.17
Mean Heart Dose	1.34 Gy (0.72)	2.45 Gy (1.9)	***p*** **= .0075**
Heart V2.5Gy	7.9%(10.2)	19.51% (12.12)	***p*** **= .0022**
Heart V10	15.88%(9.2)	16.4 (14.8)	*p* = 0.41

*SD* Standard deviation

*Vx* connoted the volume of the organ receiving “x” dose

### 4D CT data

Differences between the stability due to respiratory motion of the heart were seen between CPAP and no CPAP scans. Without CPAP the median excursion of the heart was 111 cc (range 73-185 cc). This was compared to a median excursion of 81 cc (range 37-101 cc) with the use of CPAP. The implication is that use of CPAP reduced respiratory excursion and made the location of the structures at risk more stable and consistent with the treatment planning.

### Cardiac displacement

**Fig. 2:**
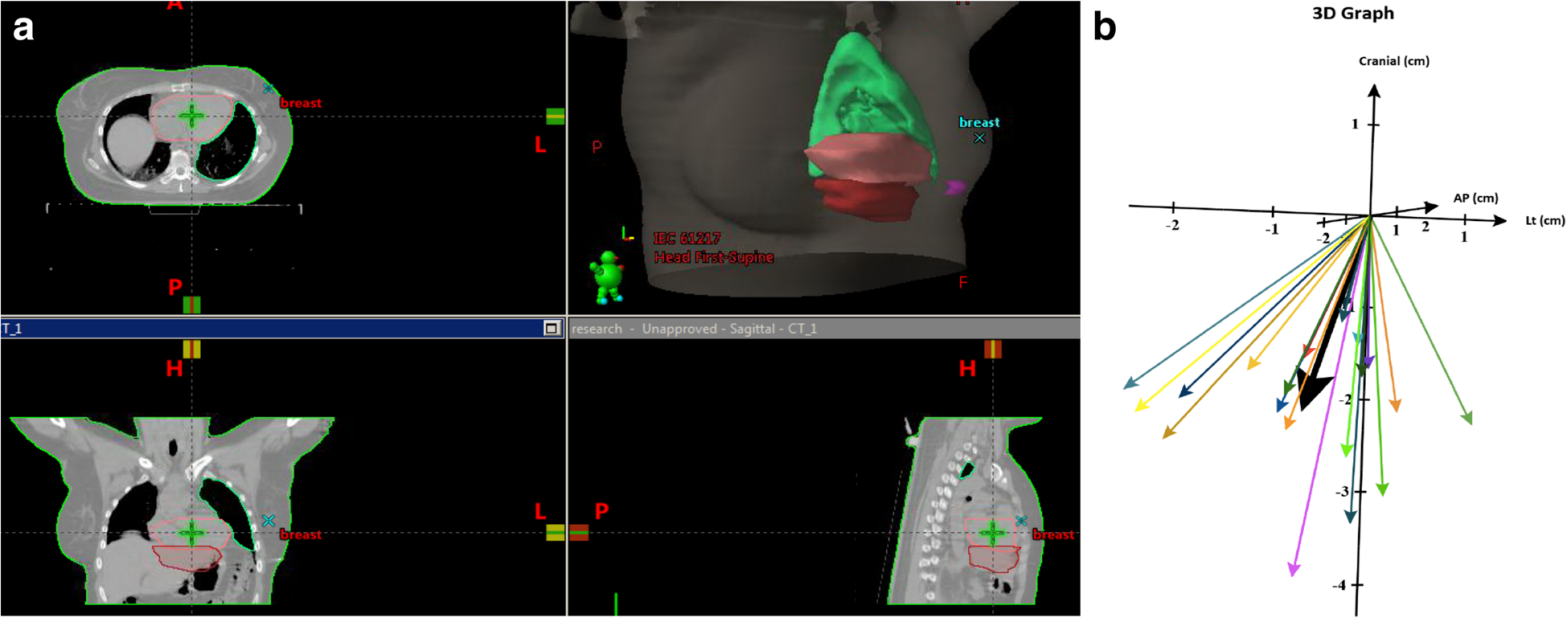
Heart Motion with CPAP. **a** depicts the cardiac location with (red) and without (peach) CPAP in one patient. One can see the caudal and inferior displacement. **b** shows a 3D- plot of the vectors for the change in center of mass of the heart with and without CPAP for each patient on the study. The thick black arrow shows the mean of all the vectors

The differences in the cardiac location due to CPAP were analyzed geographically as the motion was unique. The change in the center of mass vectors between the heart with and without CPAP is shown graphically in Fig. 2. The mean vector is also seen and shows a displacement with CPAP consistently caudally and to the right and away from the radiotherapy field.

## Discussion

The use of CPAP is well understood as a therapeutic modality for sleep apnea as well as pediatric pulmonary diseases [7, 8]. However, until recently it has not been used in the setting of radiotherapy. Goldstein and colleagues published a pioneering study detailing the potential benefits of CPAP in radiotherapy. Their study detailed the benefit of CPAP when added to SBRT for lung lesions. They demonstrated significant differences in decrease of tumor motion as well as the increase of total lung volume and decrease in both lung and heart doses [6]. A second group reported a case report of use of CPAP for left breast cancer that was unable to be planned utilizing traditional methods [9].

This small prospective trial is the first effort to our knowledge to describe the effects of CPAP on the radiotherapy of left breast cancer patients. We have shown that CPAP has the ability to significantly decrease the mean heart dose and increase the lung volume. In addition, the use of CPAP produces heart displacement caudally leading to improved dosimetry.

As stated earlier the current standard of care in many centers for left sided breast cancer is DIBH and/or IMRT. How does this standard compare to our study of CPAP?

Improving the geometry of the thorax through manipulating respiration has been very successful at reducing the cardiac dose in breast radiotherapy. The primary method that has been used is deep inspiration breath hold or DIBH. This method utilizes voluntary inflation of the chest to separate the heart from the breast. DIBH has been used and studied in multiple publications and has become a standard of care for left sided breast radiotherapy [1, 5]. The benefit of DIBH varies throughout the literature but it typically serves to reduce the mean heart dose by 50% and also significantly reduces the V20 of the ipsilateral lung [10, 11].

In our study we saw 47% reduction in mean heart dose which is comparable to DIBH studies. However, our reduction in ipsilateral V20 was only 12% on average, which is lower that what is seen in DIBH. This may be due to the small percentage of patients in our study with nodal irradiation or due to small sample size.

One challenge with DIBH is the reproducibility of the location of both the breast and the normal structures. Most breast plans delivery requires multiple respiratory cycles, which can introduce a critical level of error [12, 13]. In addition, in order to complete the treatment, it requires a cooperative and able patient. This can disqualify older patients or those with limited compliance. Finally, in centers without 4-D CT and or gated linacs this approach is possible but more cumbersome. In our study we found that CPAP improved stability of the breast CTV because it “fixes” the lung in steady state. Although our comparison was to free breathing treatment it would be interesting in future studies to compare DIBH treatment to CPAP based therapy.

One novel aspect of our study, which is unique to CPAP assisted therapy, is the change in size and location of the heart with CPAP. As shown in Fig. 2 the heart typically not only moves away from the breast and chest wall but also is pushed inferiorly. This trajectory has important implications for radiotherapy of breast cancer but also could significantly influence SBRT and other thoracic radiotherapy techniques.

Our study has clear limitations. It is a small single institution trial. There is heterogeneity of patients and plans. This is true of all series of breast cancer plans as one size does not fit all. However, our technique of using each patient as its own control does provide comparable data. In addition, the clinical benefit of this sparing remains unclear as the effects of small doses of radiation on the heart and lung are only seen years after treatment. Nevertheless, this technique shows promise and is certainly worthy of further investigation Table 2.

**Table 2:** Use of CPAP for Left Breast Radiotherapy

Pro	Con
Decreased heart dose	Contraindication with latex allergy
Improved stability of tumor volume	Requires pretreatment preparation time
Well tolerated	
Applicable to any clinical setting with minimal cost	

## Data Availability

The datasets during and/or analyzed during the current study available from the corresponding author on reasonable request.
